# Adaptation and validation of the Cambridge Pulmonary Hypertension Outcome Review (CAMPHOR) for the Netherlands

**DOI:** 10.1007/s12471-016-0849-z

**Published:** 2016-05-19

**Authors:** M. Wapenaar, J. Twiss, M. Wagenaar, P. Seijkens, L. van den Toorn, J. Stepanous, A. Heaney, A. van den Bosch, K. A. Boomars

**Affiliations:** 1 Department of Pulmonary Medicine, Erasmus Medical University Center, Rotterdam, The Netherlands; 2 Galen Research Ltd, Manchester, UK; 3 Department of Pulmonary Medicine, VU University Medical Center, Amsterdam, The Netherlands; 4 Department of Cardiology, Erasmus Medical University Center, Rotterdam, The Netherlands

**Keywords:** Pulmonary arterial hypertension, Chronic thromboembolic pulmonary hypertension, CAMPHOR health questionnaire, Health-related quality of life

## Abstract

**Background:**

The Cambridge Pulmonary Hypertension Outcome Review (CAMPHOR) is the first disease-specific instrument for pulmonary arterial hypertension (PAH) to assess patient-perceived symptoms, activity limitations and quality of life. To be able to use this questionnaire in the Netherlands, the aim of the study was to translate and validate this instrument for the Dutch-speaking population.

**Methods:**

First the CAMPHOR was translated into Dutch (by means of a bilingual and a lay panel) and field-tested by means of cognitive debriefing interviews with ten PAH patients. For psychometric evaluation, 80 patients with PAH or chronic thromboembolic pulmonary hypertension (CTEPH) were asked to complete the CAMPHOR twice over a two-week period. To test for construct validity, participants also completed the Nottingham Health Profile (NHP).

**Results:**

The Dutch version of the CAMPHOR showed high internal consistency for all scales (Cronbach’s alpha 0.89–0.91) and excellent reproducibility over two weeks (reliability coefficients 0.87–0.91). Concurrent validity showed that the CAMPHOR scales correlated as expected with the NHP scales. The CAMPHOR was able to distinguish between patient groups based on self-reported general health status, disease severity and NYHA classification demonstrating evidence of known group validity. The CAMPHOR activity limitations scale correlated moderately with the distance walked during the 6‑minute walk test (r = −0.47, *p* < 0.01) and the symptoms scale with the Borg dyspnoea score (r = 0.51, *p* < 0.01).

**Conclusion:**

The Dutch version of the CAMPHOR is a reliable and valid measure of quality of life and health status in patients with PAH and CTEPH is recommended for use in routine care and in clinical research.

## Background

Pulmonary arterial hypertension (PAH) is a progressive disease of the pulmonary vasculature, leading to increased pulmonary vascular resistance ultimately resulting in right heart failure and premature death [1]. PAH can affect persons of all ages, and females are more affected than males [1, 2]. Symptoms include breathlessness, fatigue, chest pain, palpitations, ankle oedema and syncope [1]. Unfortunately, it is not possible to cure the disease with the currently available treatments. The aim of therapy is to lengthen survival time, to ameliorate symptoms, to improve quality of life and to reduce the number of hospitalisations [3]. Chronic thromboembolic hypertension (CTEPH) is a form of precapillary PH. Patients with non-operable CTEPH suffer from the same symptoms as patients with PAH and despite treatment with specific PAH medication have a poor life expectancy [4, 5].

In spite of the current treatment options, health-related quality of life (HRQL) is impaired in most patients suffering from PAH [6–9]. HRQL should be measured with an appropriate questionnaire [10]. Generic HRQL measures employed in PAH populations are of limited value in the assessment of PAH, since these do not take into account all aspects of the disease and its treatment [11–14]. Therefore, a disease-specific outcome measure for patients with PAH has been developed, the Cambridge Pulmonary Hypertension Outcome Review (CAMPHOR) [15]. The questionnaire comprises three scales that assess overall symptoms (25 items), activity limitations (15 items) and quality of life (15 items). This tool is designed for use in clinical practice as well as clinical trials. This questionnaire has been used as an outcome parameter in studies concerning PAH over the last years [16–18]. The CAMPHOR health questionnaire has been translated and validated in several languages for several countries [19–23]. This paper describes the adaptation and the validation of CAMPHOR for Dutch-speaking population in the Netherlands.

## Methods

The adaptation of the CAMPHOR questionnaire was conducted in two PH centres in the Netherlands, the Erasmus University Medical Center in Rotterdam and the VU University Medical Center in Amsterdam. The process consisted of three stages: translation (by means of a bilingual and a lay panel), cognitive debriefing interviews with ten PAH patients and validation by means of a postal validation study. The study was approved by the ethics committees of both centres.

## Translation process

A professional translation panel of six individuals who were fluent in both English and Dutch, led by the local investigator’s representative and a research scientist from Galen Research, produced the first translation. A separate lay panel consisting of five individuals of average educational level (3 men and 2 women aged between 21 and 67 years) discussed the proposed wording of the items and decided whether these were acceptable or required adjustments to improve the clarity and to make the sentences sound more natural. The local investigator evaluated and discussed the changes made with the scientist from Galen Research.

### Cognitive debriefing interviews

The draft version of the instrument was tested with ten patients, via one-to-one semi-structured interviews. A representative selection of PAH patients was made based on gender, age, severity of PAH and social background. The respondents were asked to complete the questionnaire in the presence of an interviewer who observed whether any problems were experienced. Respondents were required to provide feedback on their comprehension of the measure and the relevance of the items.

### Postal validation survey

During a consecutive three-month period from September 2014 to December 2014, 80 Dutch-speaking patients (who were able to read the Dutch language), suffering from pre-capillary PAH (WHO group 1) or CTEPH (WHO group 4), were asked to complete the new language version of the CAMPHOR and the Nottingham Health Profile (NHP) on the day of their clinic visit and the CAMPHOR questionnaire again after two weeks.

The NHP is a generic measure of perceived distress consisting of 38 items divided into six sections (energy level, pain, emotional reactions, sleep, social isolation and physical ability) [24, 25]. In both questionnaires higher scores indicate worse health status.

Baseline characteristics were obtained (sex, age, employment status) and illness information (duration of PH, perceived general health, self-perceived disease severity, oxygen use) was also collected. The NYHA functional class was determined, a six-minute walk test (6MWT) was performed and the NT-pro BNP level was measured.

Patients were asked to complete the questionnaires at home and to return the questionnaires by post in pre-addressed, stamped envelopes. After two weeks, they received a phone call to remind them to fill in the second CAMPHOR questionnaire and to inquire about possible changes in their physical health.

### Withdrawal of patients

Patients who did not complete more than 85 % of a questionnaire were withdrawn from the analysis. For the test-retest reliability, patients were excluded if they were not clinically stable.

### Data analysis

Continuous variables are expressed as mean ± SD. Internal consistency of the CAMPHOR adaptation was evaluated by determining Cronbach’s alpha coefficient. An alpha coefficient >0.7 is considered to be the minimum value required to indicate sufficient internal consistency.

Test re-test reliability (patient-specific agreement between two repeated administrations) was examined using Spearman’s rank correlations. Correlation coefficients above 0.85 indicate good reproducibility [26]. Convergent validity was assessed with the NHP as the comparator instrument using Spearman’s rank correlations. Known group validity was tested by Mann-Whitney U test. Correlation between CAMPHOR scores, demographic factors, the results of the 6MWT (including Borg scores) and NT-pro BNP levels was assessed using Spearman’s rank correlations. A p-value of <0.05 was considered statistically significant.

## Results

### Bilingual panel

The group reached consensus on the appropriate wording for most items. A few phrases could not be translated literally. For example, one item from the symptoms scale; ‘My stamina levels are low’ was translated as ‘Mijn lichamelijke conditie is slecht’ (literally: ‘My physical condition is weak’). For a few items consensus could not be reached and alternative versions of these items were taken forward for consideration by the lay translation panel.

### Lay panel

Some expressions were altered from the original translation into more commonly used Dutch. For example, for item 9 of the symptoms scale: ‘I soon run out of energy’. This sentence was translated as; ‘Mijn energie is snel op’. The panel felt that this translation was too literal. They instead proposed: ‘Ik heb weinig energie’ (literally: ‘I have little energy’).

### Cognitive debriefing interviews

Ten patients were interviewed (6 females, 4 males, mean age 49.1, range 20–77 years, PH symptoms ranged from mild to quite severe). Average time for completion was 12.6 minutes (range 6–24 minutes, median 11.5 minutes). Overall patients thought the questionnaire was appropriate and applicable. Some patients found it hard to choose between the ‘Yes’ or the ‘No’ response format, and would have liked the option of ‘Sometimes’. For the activity limitations scale, the response option ‘Doing it on your own with problems’ was changed into ‘With difficulties doing it on your own’. In the quality of life section item 17; ‘I feel that I’m losing my role in life’, translated as; ‘Ik voel dat ik mijn rol(len) [verantwoordelijkheden] in het leven verlies’ was considered to be a difficult question by the majority of the patients.

### Postal validation survey

From the 80 patients who were asked to participate, 76 completed and returned the questionnaires. Of these only 0.14 % of the items were missing. Missing items from the CAMPHOR as well as the NHP questionnaire were handled according to the manuals. Demographic and disease characteristics of the respondents are listed in Tab. 1. The cohort consisted of 59 females and 17 males, which is consistent with the gender ratio in a PAH population. Disease information is listed in Tab. 2. The descriptive statistics for the questionnaires at both time points are shown in Tab. 3. High floor effects (high number of patients scoring the minimum) were observed in the NHP subscales, but not in the CAMPHOR scales.

**Tab. 1:** Demographic and patient characteristics

Characteristics		Patients (*n* = 76)	Percentage (%)
Sex	Male	17	22.3
	Female	59	77.7
Age in years	Mean	56	
	Median	59.5	
	Range	20–79	
Diagnosis in years	Mean	7.1	
	Median	4.2	
	Range	0–50	
Aetiology	IPAH	26	34.2
	HPAH	4	5.3
	Congenital heart disease	5	6.6
	Connective tissue disease	11	14.5
	HIV	3	3.9
	Porto pulmonary	3	3.9
	PVOD	1	1.3
	Other	3	3.9
	CTEPH	20	26.3
NYHA classification	1	0	0
	2	56	73.7
	3	20	26.3
	4	0	0
Treatment	ERA monotherapy	13	17.1
	PDE-5 inhibitor monotherapy	7	9.2
	Riociguat	2	2.6
	Duo therapy: ERA and PDE-5 inhibitor	30	39.5
	Prostacyclin monotherapy	6	7.9
	Prostacyclin and PDE-5 inhibitor	2	2.6
	Prostacyclin and ERA	1	1.3
	Triple therapy prostacyclin, ERA and PDE-5 inhibitor	11	14.5
Require oxygen	No Yes	61 14	81.3 18.7
6-minute walking distance in meters	Mean	466	
	Median	472	
	Range	232–647	
	Missing	4	
NT-proBNP in pmol/ml	Mean	53.4	
	Median	24.8	
	Range	3.9–439.2	

*IPAH* idiopathic pulmonary arterial hypertension, *HPAP* heritable pulmonary arterial hypertension, *PVOD* pulmonary veno-occlusive disease, *CTEPH* chronic thromboembolic pulmonary hypertension, *ERA* endothelin receptor antagonist, *PDE-5 inhibitor* phosphodiesterase-5 inhibitor

**Tab. 2:** Disease information at time 1 (*n* = 76)

	Number of patients	Percentage (%)
**Self-reported general health**		
Poor	6	7.9
Fair	32	42.1
Good	32	42.1
Very good	6	7.9
**Self-reported severity of disease**		
No symptoms	8	10.7
Mild	28	37.3
Moderate	35	46.7
Quite severe	3	4.0
Very severe	1	1.3
**Flare up**		
No	72	94.7
Yes	4	5.3

**Tab. 3:** Questionnaire descriptive statistics

	***n***	**Median (IQR)**	**Mean (SD)**	**Min–Max**	**% scoring minimum**	**% scoring maximum**
**Time 1**						
CAMPHOR symptoms	76	4.0 (2.0–8.0)	5.3 (4.6)	0.0–25.0	13.2	0.0
CAMPHOR activities	76	4.0 (2.0–9.0)	5.6 (4.9)	0.0–30.0	14.5	0.0
CAMPHOR QoL	76	4.0 (1.0–8.0)	5.1 (4.9)	0.0–25.0	14.5	0.0
**NHP**						
Energy scale	74	0.0 (0.0–33.3)	19.8 (32.6)	0.0–100.0	66.2	9.5
Pain scale	75	0.0 (0.0–0.0)	7.0 (18.4)	0.0–100.0	78.7	0.0
Emotional Reactions	75	0.0 (0.0–11.1)	10.8 (18.7)	0.0–100.0	58.7	1.3
Sleep scale	75	20.0 (0.0–40.0)	25.3 (30.2)	0.0–100.0	46.7	2.7
Social isolation	74	0.0 (0.0–0.0)	5.1 (13.7)	0.0–100.0	85.1	0.0
Physical mobility	74	12.5 (0.0–25.0)	15.4 (19.0)	0.0–100.0	47.3	0.0
NHP–D	73	2.0 (0.0–4.0)	2.9 (3.9)	0.0–24.0	32.9	0.0
**Time 2**						
CAMPHOR Symptoms	74	6.0 (1.8–9.0)	5.9 (5.0)	0.0–25.0	16.2	0.0
CAMPHOR Activities	75	4.0 (2.0–9.0)	5.9 (5.1)	0.0–30.0	17.3	0.0
CAMPHOR QoL	74	3.0 (1.0–8.3)	4.9 (5.2)	0.0–25.0	21.6	0.0

### Internal consistency

For all three CAMPHOR scales, Cronbach’s alpha coefficients were above 0.8, indicating high internal consistency (detailed in Tab. 4).

**Tab. 4:** Cronbach’s alpha coefficients

	**Time 1**	**Time 2**
CAMPHOR symptoms	0.89	0.89
CAMPHOR activities	0.91	0.90
CAMPHOR QoL	0.89	0.91

### Test-retest reliability

Test-retest reliability was excellent for all three scales, (0.87 for symptoms, 0.91 for activity and 0.87 for quality of life), which demonstrates low levels of random measurement error.

### Convergent validity

The CAMPHOR symptoms scale correlated strongly with the energy and physical mobility scales of the NHP, showing the importance of these factors on PAH symptomatology. It also correlated moderately with Borg dyspnoea scores. There were significant correlations between the CAMPHOR QoL scale and the NHP energy scale, physical mobility and NHP-D (summation of sub-set of NHP items scores) indicating that multiple factors influence QoL. As expected, the activity limitations scale showed the strongest correlation with the NHP physical mobility and 6MWT. The correlation coefficients between CAMPHOR scales and the NHP are listed in Tab. 5.

**Tab. 5:** Correlation coefficients between CAMPHOR scales and NHP, 6MWT and NT-proBNP

	Symptoms	Activities	QoL
*NHP*			
Energy scale	0.71*	0.65*	0.66*
Pain scale	0.38*	0.38*	0.42*
Emotional reactions	0.43*	0.24**	0.37*
Sleep scale	0.32*	0.22	0.38*
Social isolation	0.23	0.34*	0.39*
Physical mobility	0.67*	0.76*	0.61*
NHP-D	0.58*	0.49*	0.63*
*6MWT*			
Distance walked(m)	−0.34*	−0.47*	−0.42*
Borg dyspnoea score	0.51*	0.49*	0.32*
NT-proBNP	−0.08	−0.08	0.10

Values shown represent Spearman’s rank correlation coefficients

*p < 0.01; **p < 0.05

No significant correlations were demonstrated between the CAMPHOR scales and the NT-pro BNP (Tab. 5).

### Association of CAMPHOR scores and demographic factors

No significant differences in the CAMPHOR scores were found between patients grouped by age. However, significant differences were shown in the scores of symptoms and QoL scales between males and females. Females scored higher on these two scales compared with males. A chi-square test of independence was performed to assess the relation between gender and self-reported severity of disease. No significant association was found between these variables (χ^2^ (1, *n* = 75) = 0.08, *p* = 0.93). Similarly, no significant relationship was found between gender and NYHA class (χ^2^ (1, *n* = 76) = 1.1, *p* = 0.74). The relation between gender and cause of PH was also investigated, but again no significant association was found (χ^2^ (7, *n* = 76) = 8.5, *p* = 0.29).

### Known group validity

**Fig. 1:**
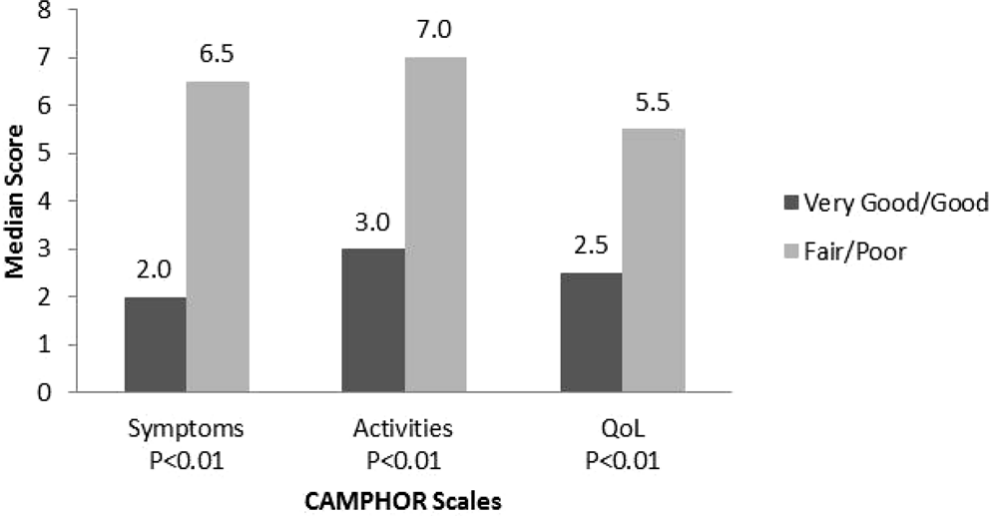
*Median CAMPHOR scales scores for self-reported general health tested with Mann Whitney U*. Interquartile ranges (IQR) for the Camphor scales scores for very good/good and fair/poor, respectively, are: Symptoms 0.8–4.3 and 4.0–11.0; Activities 1.0–5.0 and 3.0–10.0; Qol 1.0–5.0 and 1.8–10.0

**Fig. 2:**
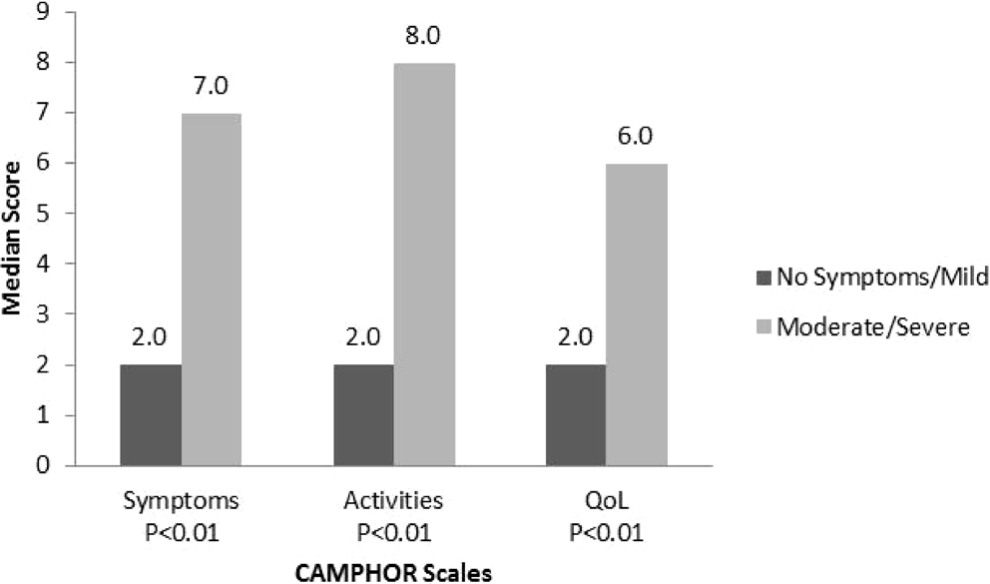
Median CAMPHOR scales scores for self-reported disease severity tested with the Mann-Whitney U test. Interquartile ranges (IQR) for the Camphor scales scores for no symptoms/mild and moderate/severe, respectively, are: Symptoms 0.0–3.0 and 5.0–11.0; Activities 0.3–5.8 and 3.0–10.0; Qol 0.0–5.0 and 3.0–10.0

CAMPHOR scales scores were able to discriminate between patients based on perceived general health (‘very good/good’ versus ‘fair/poor’) and severity of disease (‘no symptoms/mild’ versus ‘moderate/severe’). Patients with worse perceived general health (Fig. 1) and more severe PAH (Fig. 2) had higher scores for all three scales of the CAMPHOR.

**Fig. 3:**
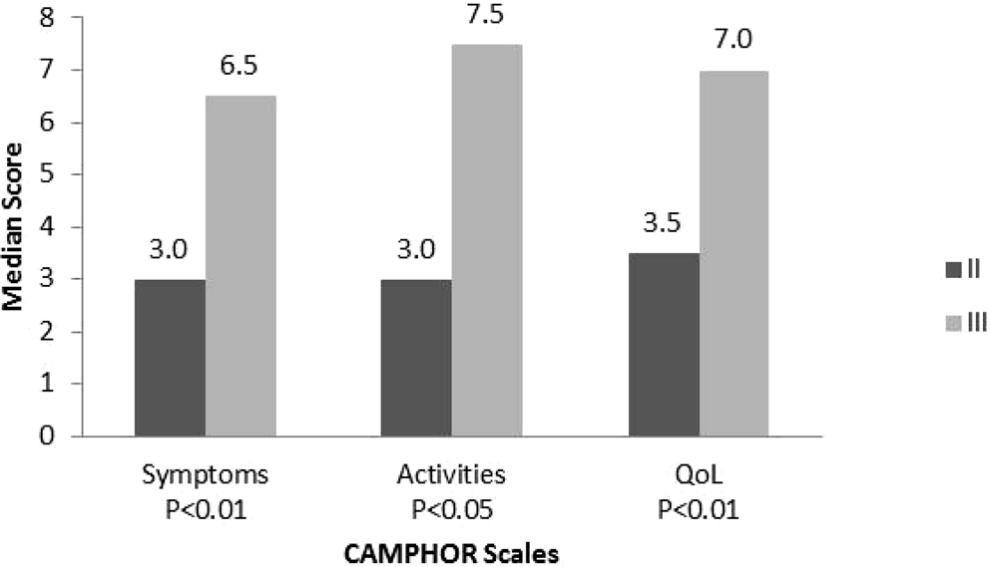
Median CAMPHOR scores and NYHA classification tested with the Mann-Whitney U test. Interquartile ranges (IQR) for the Camphor scales scores for class II and class III, respectively, are: Symptoms 1.0–7.0 and 4.0–11.8; Activities 2.0–8.0 and 3.0–12.5; Qol 1.0–5.8 and 2.3–11.5

Patients in NYHA class 3 showed significantly higher scores on all three CAMPHOR scales compared with patients in NYHA class 2 (Fig. 3).

**Fig. 4:**
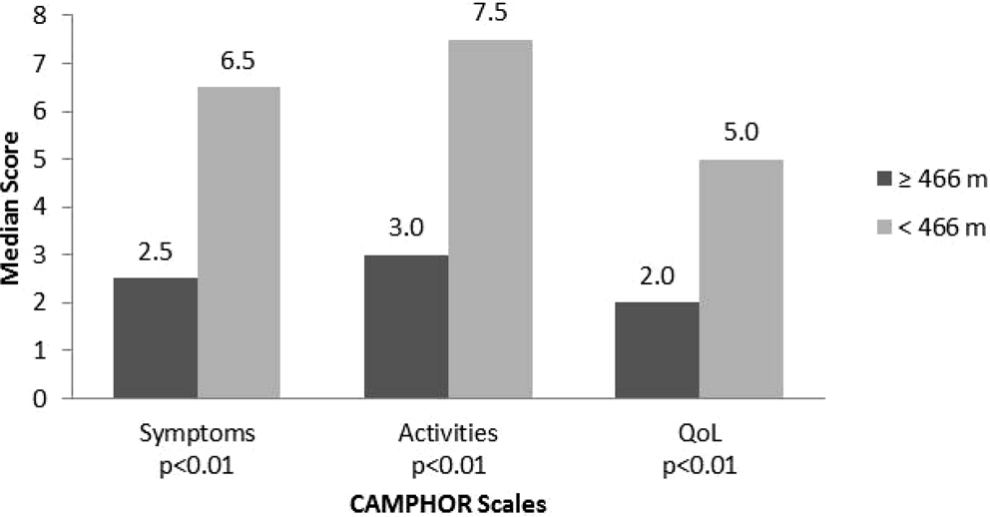
Group validity of six-minute walk distance ≥ 466 m and < 466 m and median CAMPHOR scales scores using the Mann-Whitney U test. Interquartile ranges (IQR) for the Camphor scales scores for ≥466 m and 466 m, respectively, are: Symptoms 1.0–3.0 and 3.0–9.8; Activities 1.0–5.0 and 3.0–12.8; Qol 0.0–5.8 and 4.0–8.0

Patients grouped based on the distance walked during 6MWT (below and above the mean value of 466 metres) showed significant differences in all CAMPHOR scales (Fig. 4).

No differences were observed in the CAMPHOR subscales between PAH and CTEPH patients (16 patients in NYHA class 2 and four patients in NYHA class 3), tested by the Mann-Whitney U test: CAMPHOR symptoms *p* = 0.59, CAMPHOR activities *p* = 0.92 and CAMPHOR quality of life *p* = 0.94.

## Discussion

This study demonstrates that the new adaptation of the CAMPHOR for Dutch-speaking participants in the Netherlands is valid and reliable. The objective of adapting the questionnaire is to ensure that items are understood in the same way in different countries and that conceptual equivalence rather than linguistic equivalence is achieved in the translated items. Moreover, it is vital that translated items are expressed in common (everyday) language. No major problems were encountered during the translation process.

Descriptive statistics showed the CAMPHOR had low floor effects and no ceiling effects, which indicates the CAMPHOR is well targeted to the PAH population. Consequently, the measure should be sensitive and responsive in clinical studies (e. g. in longitudinal studies). In contrast, the NHP showed very high floor effects indicating patients with the lowest possible score cannot be distinguished from each other, which reduces sensitivity.

Cronbach’s alpha coefficients were above 0.8 for the three CAMPHOR scales, indicating that the items were related adequately to form scales. Test-retest reliability was excellent for all three scales showing the scales have low levels of random measurement error.

The CAMPHOR scales showed different levels of association with the scales of the NHP, demonstrating evidence of convergent validity. As expected, CAMPHOR activities correlated most strongly with the NHP physical mobility scale and 6MWT as was also shown by Cima et al. in the German adaptation of the CAMPHOR [22].

Patients with worse perceived general health and more severe PAH had higher scores for all three scales of the CAMPHOR scores showing that the scales could distinguish appropriately between groups of known importance.

Females scored higher on the scales of symptoms and QoL compared with males. Further analyses were performed to investigate this difference. The relation between gender and self-reported severity of disease as well as gender and NYHA class and gender and cause of PAH was assessed. No significant association was found between gender and the investigated variables. Based on these findings it was unclear what contributed to the differences between gender groups. However, due to the relatively small sample of males the results could be spurious.

The sample of patients included in this study seemed to have less severe disease than the sample included in the original paper describing the development of the CAMPHOR questionnaire. One explanation may be that with the currently available treatment, including triple therapy, less patients are now in NYHA class 4. Another explanation might be that only patients who visited the outpatient clinic were asked to participate in the study. In this way the very severe patients, who were hospitalised during this period (for example those waiting for lung transplantation), were not included.

However, the CAMPHOR scores were able to clearly distinguish between patients in NYHA class 2 and NYHA class 3. Moreover, the results of the 6MWT correlate well with the CAMPHOR scale scores.

## Conclusions

The new Dutch language version of the CAMPHOR is a valid and reliable instrument for assessment of health-related quality of life in PAH and CTEPH patients and is recommended for use in clinical practice. Moreover the CAMPHOR provides a valid tool for a single-point measurement in cross-sectional studies.
