# Correction to: Adaptation and validation of the Cambridge Pulmonary Hypertension Outcome Review (CAMPHOR) for the Netherlands

**DOI:** 10.1007/s12471-018-1158-5

**Published:** 2018-10-26

**Authors:** M. Wapenaar, J. Twiss, M. Wagenaar, P. Seijkens, L. van den Toorn, J. Stepanous, A. Heaney, A. van den Bosch, K. A. Boomars

**Affiliations:** 1 Department of Pulmonary Medicine, Erasmus Medical University Center, Rotterdam, The Netherlands; 2 Galen Research Ltd, Manchester, UK; 3 Department of Pulmonary Medicine, VU University Medical Center, Amsterdam, The Netherlands; 4 Department of Cardiology, Erasmus Medical University Center, Rotterdam, The Netherlands

**Correction to:**

**Neth Heart J 2016**

10.1007/s12471-016-0849-z

Unfortunately the original version of this article contained Electronic Supplementary Material which should not have been published with the article due to copyright reasons.

The original version has been updated and the ESM has been removed.

